# Intralesional triamcinolone versus 5-fluorouracil for keloids and hypertrophic scars: A randomized comparative clinical study

**DOI:** 10.6026/973206300220461

**Published:** 2026-01-31

**Authors:** Anubhav Sharma, Thirumalaiswamy T, Amulya Singirikonda, Prameela K, Supritha Patil B, Alekhya Achanta, Shan Nawaz Malik

**Affiliations:** 1 Department of Orthopedics, Dayanand Medical College & Hospital, Ludhiana, Punjab, India; 2 Department of General Surgery, Dhanalakshmi Srinivasan Medical College & Hospital, Siruvachur, Chennai-Trichy National Highway, Perambalur, Tamil Nadu, India; 3 Department of dental surgery, Mamata Dental College and Hospital, Khammam, Telangana, India; 4 Department of Oral Medicine and Radiology, Mallareddy Dental College for Women, Malla Reddy Vishwavidyapeeth, Suraram, Hyderabad, Telangana, India; 5 Department of Oral Medicine and Radiology, Tirumala Institute of Dental Sciences and Research Centre, Nizamabad, Telangana, India; 6 Department of Paediatric and Preventive Dentistry, Malla Reddy Institute of Dental Sciences, Malla Reddy Vishwavidyapeeth, Suraram, Hyderabad, Telangana, India; 7 Department of Dentistry, Dental Clinician, London, United Kingdom

**Keywords:** Keloid, hypertrophic scars, intralesional injection, triamcinolone acetonide, 5-fluorouracil

## Abstract

Keloids and hypertrophic scars lack a consistently effective and safe intralesional treatment and direct randomized comparisons between triamcinolone acetonide and 5-fluorouracil remain limited. Therefore, it is of interest to randomize clinical trial compared both agents in 100 patients treated over 12 weeks, evaluating scar height reduction, symptom improvement and adverse effects. Both treatments achieved significant scar flattening, with triamcinolone showing marginally greater mean height reduction. However, triamcinolone was associated with substantially higher rates of skin atrophy and telangiectasia, while 5-fluorouracil demonstrated a superior safety profile. Thus, we show 5-fluorouracil as a suitable first-line option for keloids and hypertrophic scars, especially in cosmetically sensitive areas.

## Background:

Keloids and hypertrophic scars are abnormal wound-healing responses marked by excessive fibroblast activity and collagen deposition beyond original wound margins. These lesions cause cosmetic deformity, pruritus, pain and psychological distress. Current treatment options include intralesional corticosteroids, silicone sheets, pressure therapy, cryotherapy, laser therapy, radiotherapy and surgical excision, yet recurrence remains common [1, 2- 3]. Intralesional triamcinolone acetonide is widely used due to its anti-inflammatory and collagen-suppressive effects. However, steroid therapy frequently produces skin atrophy, hypopigmentation and telangiectasia and many keloids show partial or complete steroid resistance [4]. The antimetabolite 5-fluorouracil inhibits fibroblast proliferation and collagen gene expression, making it a promising alternative treatment [5, 6-7]. Meta-analyses indicate that combining triamcinolone and 5-fluorouracil improves outcomes and reduces complications compared to steroid monotherapy [8, 9-10]. Despite this, randomized direct comparisons between triamcinolone and 5-fluorouracil monotherapy remain limited. Therefore, it is of interest to a randomized comparative clinical study was undertaken to evaluate their efficacy and safety in keloids and hypertrophic scars.

## Materials and Methods:

This single-centre, randomized, parallel-group clinical study enrolled 100 patients aged 18-60 years presenting with clinically diagnosed keloids or hypertrophic scars of ≥6 months duration and scar height ≥2 mm. Exclusion criteria included pregnancy/lactation, uncontrolled diabetes, hepatic or renal dysfunction, active infection at scar site, prior intralesional therapy within 3 months and immunocompromised status. After obtaining informed consent, participants were randomized 1:1 to Group A (intralesional TAC, 40 mg/mL, injection every 3 weeks) or Group B (intralesional 5-FU, 50 mg/mL, injection every 3 weeks). Treatments were administered for up to 4 sessions (12 weeks). At baseline and at weeks 3, 6, 9 and 12, measurements were recorded for scar height (in mm, using callipers), Vancouver Scar Scale (VSS) parameters (height, vascularity, pliability, pigmentation), pruritus and pain (by 0-10 visual analogue scale, VAS) and adverse events (skin atrophy, telangiectasia, ulceration, hypopigmentation). Primary endpoint was mean reduction in scar height at week 12. Secondary endpoints included improvement in VSS and symptom scores and incidence of adverse effects. Statistical analysis used independent t-tests for continuous variables and chi-square for categorical variables; p < 0.05 was considered significant.

## Results:

Both treatment arms demonstrated significant reductions in scar height after 12 weeks, indicating overall therapeutic benefit. The triamcinolone group achieved a slightly higher mean reduction compared with the 5-fluorouracil group, suggesting stronger efficacy in flattening lesions. Baseline parameters such as age, sex distribution and scar duration were comparable between groups, ruling out demographic bias. The statistical difference (p = 0.03) confirms a modest superiority of triamcinolone in height reduction (Table 1). Both groups showed noticeable improvement in Vancouver Scar Scale scores and symptom relief. While pruritus and pain decreased similarly in each arm, triamcinolone yielded greater reduction in VSS total score. However, adverse reactions like skin atrophy and telangiectasia were substantially more frequent with triamcinolone injections compared with 5-fluorouracil. No significant difference in ulceration rates was seen, underscoring a better safety profile of 5-fluorouracil (Table 2).

## Discussion:

This randomized comparative study evaluated intralesional triamcinolone acetonide and 5-fluorouracil in keloids and hypertrophic scars over 12 weeks. Triamcinolone produced slightly greater scar height reduction and Vancouver Scar Scale improvement. However, local adverse effects such as skin atrophy and telangiectasia were significantly higher with triamcinolone. These findings are consistent with controlled trials showing effective scar flattening but inferior safety with corticosteroid therapy [11, 12]. Triamcinolone suppresses fibroblast activity through anti-inflammatory pathways, while 5-fluorouracil inhibits fibroblast DNA replication and collagen synthesis, resulting in reduced fibrosis with minimal tissue damage [13]. Previous studies report superior long-term outcomes when both agents are combined, although monotherapy comparisons remain scarce [14, 15]. The small difference in height reduction observed in this study is unlikely to provide substantial cosmetic advantage. In contrast, the higher complication rate with triamcinolone directly affects patient satisfaction and aesthetic outcome. Reviews indicate that 5-fluorouracil offers better tolerability and adherence in clinical practice [17, 18]. Newer delivery methods such as laser-assisted and jet-injection techniques further improve drug distribution and reduce adverse events [19]. The short follow-up period limits recurrence assessment, which remains a major challenge in keloid therapy [20]. Longer follow-up trials with combination arms are recommended. Based on safety and efficacy balance, 5-fluorouracil is better suited for cosmetically sensitive areas, while triamcinolone remains useful where rapid flattening is prioritized. This study provides randomized head-to-head evidence comparing triamcinolone and 5-fluorouracil monotherapy in keloids and hypertrophic scars. It demonstrates that marginally higher flattening efficacy of triamcinolone is offset by significantly greater adverse effects. The findings support 5-fluorouracil as a safer first-line alternative in cosmetically sensitive regions. This directly addresses the existing gap in comparative clinical evidence.

## Conclusion:

The intralesional TAC achieved marginally high scar height reduction in keloids/hypertrophic scars. However, it was associated with a significantly high rate of local adverse events compared with 5-FU. Thus, 5-FU is a viable alternative for cosmetically sensitive areas or patients with heightened concern for steroid-related complications. Future larger trials with longer follow-up and combination arms are warranted.

## Figures and Tables

**Table 1:** Baseline characteristics and scar height reduction at week 12

**Parameter**	**Group A (TAC, n=50)**	**Group B (5-FU, n=50)**	**p-value**
Mean age (years)	34.2±10.1	33.8±9.5	0.82
Male : Female ratio	28:22:00	26:24:00	0.69
Scar duration (months)	14.5±6.2	13.9±5.8	0.55
Baseline scar height (mm)	4.8±1.2	4.7±1.3	0.68
Reduction in scar height at week 12	2.1±0.9	1.8±0.8	0.03

**Table 2:** Symptom improvement and adverse effects at week 12

**Outcome**	**Group A (TAC)**	**Group B (5-FU)**	**p-value**
Mean VSS score reduction	4.2±1.1	3.7±1.0	0.04
Pruritus VAS reduction	3.1±1.2	2.9±1.1	0.38
Pain VAS reduction	2.4±1.0	2.2±0.9	0.29
Skin atrophy incidence	22/50 (44%)	4/50 (8%)	<0.001
Telangiectasia incidence	25/50 (50%)	11/50 (22%)	0.002
Ulceration incidence	1/50 (2%)	3/50 (6%)	0.31
